# Effects of Resistance Exercise on Quality of Life, Anxiety, Depression, Sleep Quality and Inflammatory Parameters in Patients with Colorectal Cancer Undergoing Active Treatment: A Pilot Randomized Clinical Trial

**DOI:** 10.3390/curroncol32120651

**Published:** 2025-11-21

**Authors:** Juan Luis Sánchez-González, Jesus Perez, Eduardo José Fernández-Rodríguez, Emilio Fonseca-Sánchez, Yolanda López-Mateos, Claudia María Sanz-Blanco, Francisco Javier Martín-Vallejo, Alberto García-Martín, Carlos Martín-Sánchez

**Affiliations:** 1 Department of Medicine, Faculty of Medicine, University of Salamanca, 37007 Salamanca, Spain; juanluissanchez@usal.es (J.L.S.-G.); jesusperez@usal.es (J.P.); 2 Institute of Biomedical Research of Salamanca (IBSAL), 37007 Salamanca, Spain; efonseca@usal.es (E.F.-S.); yolandalopezmateos@gmail.com (Y.L.-M.); cmsanz@saludcastillayleon.es (C.M.S.-B.); jmv@usal.es (F.J.M.-V.); albergm@usal.es (A.G.-M.); carlos_ms@usal.es (C.M.-S.); 3 Department of Psychiatry, University of Cambridge, Cambridge CB2 0SZ, UK; 4 Cambridgeshire and Peterborough NHS Foundation Trust, Cambridge CB21 5EF, UK; 5 Norwich Medical School, University of East Anglia, Norwich NR4 7TJ, UK; 6 Department of Nursing and Physiotherapy, University of Salamanca, 37007 Salamanca, Spain; 7 Department of Medical Oncology, University Hospital of Salamanca, 37007 Salamanca, Spain; 8 Department of Statistics, Faculty of Medicine, University of Salamanca, 37007 Salamanca, Spain; 9 Department of Labour Law and Social Work, University of Salamanca, 37007 Salamanca, Spain

**Keywords:** colorectal cancer, drug therapy, mental health, quality of life, resistance training

## Abstract

Objective: The primary objective of this pilot randomized clinical trial was to determine the effect of adding a supervised resistance exercise programme to a home-based physical activity plan on health-related quality of life in patients with colorectal cancer undergoing active treatment. The secondary objectives were to evaluate its effects on anxiety, depression, sleep quality, and inflammatory parameters. Methods: This is a pilot randomized clinical trial with parallel groups. Patients with CRC were recruited through the Oncology Department at the Salamanca University Health Care Complex in Spain. They were randomly allocated to receive either a home-based physical activity plus a supervised resistance training programme, or the home-based physical activity plan only. The primary outcome was health-related quality of life measures and the secondary outcomes included anxiety, depression and sleep quality evaluations. The supervised training lasted 8 weeks for each patient. Results: A total of 40 patients were recruited, 20 for each group. Adding a supervised resistance exercise programme to the home-based activity plan improved symptoms related to quality of life, such as fatigue (*p* = 0.040) and constipation (*p* = 0.015). However, no significant effect was found with regard to other health-related quality of life, anxiety, depression or sleep variables. Conclusions: Fatigue and constipation in patients with CRC receiving chemo- and/or immunotherapy may benefit from the introduction of supervised resistance exercise training programmes.

## Simple Summary

People with colorectal cancer often experience fatigue, digestive problems, anxiety, and poor sleep during treatment, which can reduce their quality of life. Exercise is known to help, but it is not clear which type of exercise provides the most benefit. In this study, we tested whether adding a supervised strength training program to a simple home-based activity plan could improve the wellbeing of patients undergoing chemotherapy or immunotherapy. The results showed that supervised resistance exercise helped reduce fatigue and constipation, two of the most common and distressing symptoms. However, it did not significantly improve anxiety, depression, or sleep quality. These findings suggest that supervised strength training may play a valuable role in supportive care for patients with colorectal cancer, and they highlight the need for longer and more comprehensive exercise programs to better address the wide range of challenges faced during treatment.

## 1. Introduction

Colorectal cancer (CRC) is one of the most prevalent cancers globally and has a significant impact on the quality of life of those who suffer from it, especially when they are receiving chemotherapy [1,2]. Mortality rates from CRC have decreased in developed countries, largely due to the introduction of improved screening programmes and therapeutic advances [3]. However, the increasing number of individuals living with a current or previous diagnosis of CRC highlights the importance of appropriate supportive care to address their physical and social needs. Indeed, patients who survived CRC still experience significant physical and emotional sequelae, including anxiety, depression and sleep disorders [4,5].

The presence of pain, bowel dysfunction, and persistent fatigue directly affects these patients’ wellbeing, while the psychological impact of the diagnosis and clinical progression contributes to a high prevalence of anxiety and depressive disorders [6]. In fact, they can also present insomnia and non-restorative sleep. In turn, disruption in sleep patterns contributes to increased fatigue and worsening of depressive and anxiety symptoms, with a subsequent deterioration of their quality of life [7]. Therefore, it is essential to implement assessment and intervention strategies that address not only physical symptoms but also potential mental health, emotional and sleep problems.

Regular physical exercise has become a well-established therapeutic strategy to reduce physical symptoms and improve the quality of life of patients with CRC [8,9]. Physical exercise helps improve fatigue, improve bowel function, preserve muscle mass, and enhance overall functioning [10].

Moreover, accumulating evidence indicates that exercise exerts a beneficial effect on psychological wellbeing by reducing anxiety and depressive symptoms, both of which are known to negatively influence treatment adherence and overall prognosis in oncology. In addition, physical exercise contributes to the regulation of circadian rhythms and sleep cycles, thereby promoting restorative rest and interrupting the deleterious cycle of fatigue, emotional disturbances, and functional decline [11,12].

In this context, personalised exercise programmes, tailored to each individual’s clinical condition, may represent a promising intervention to improve physical, emotional, and social outcomes in this population [13]. Indeed, unlike conventional exercise programmes for people with CRC that only promote general physical exercise, this study introduces a new intervention that combines a structured, supervised strength training programme with a home-based physical activity plan. Flexible and supervised personalised intervention in both clinical and home settings meets the current demand for cost-effective and sustainable strategies that enhance adherence and long-term outcomes.

Against this background, the present study is designed to evaluate the effect of a hybrid, supervised physical exercise programme in patients with colorectal cancer undergoing active treatment. The primary outcome will be health-related quality of life, while secondary outcomes will comprise anxiety, depression, and sleep quality, enabling a comprehensive assessment of both physical and psychosocial benefits associated with this intervention.

Thus, the present study had the following objectives:

Primary objective: to assess the effect of a hybrid, supervised resistance exercise programme on health-related quality of life in patients with colorectal cancer undergoing active chemo- and/or immunotherapy; and Secondary objectives: to examine the impact of the intervention on anxiety, depression, sleep quality, and systemic inflammation. We hypothesized that the hybrid programme would lead to greater improvements in quality of life and reductions in fatigue and psychological distress compared to home-based activity alone.

## 2. Materials and Methods

### 2.1. Trial Design and Study Setting

A parallel-group randomised controlled trial of patients currently treated with chemo- and/or immunotherapy for CRC was conducted following CONSORT guidelines [14]. Data collection was conducted at the Salamanca Healthcare Complex (CAUSA) and the Faculty of Nursing and Physiotherapy at the University of Salamanca, Salamanca, Spain.

The study received approval from the Ethics Committee of the University of Salamanca (record number 1209) and was conducted in accordance with the Declaration of Helsinki. The clinical trial was registered in ClinicalTrials.gov (registration number NCT06404359, Registered on 8 May 2024).

### 2.2. Participants

The study recruited patients currently receiving treatment, such as chemo- and/or immunotherapy, for CRC at CAUSA’s Oncology Department, according to the following inclusion and exclusion criteria:

Inclusion criteria:

• Not having participated in any previous physical exercise programme in the last 8 weeks.
• Ability to perform physical exercise and understand evaluation questionnaires.
• Voluntary participation in the study.
• Over 18 years old.

Exclusion criteria:

• Contraindication of physical exercise, e.g., comorbid musculoskeletal diseases, severe cardiovascular disease and bone metastases, among others.
• Presence of other types of cancer than CRC.
• Early treatment discontinuation due to intolerance.

### 2.3. Intervention

The supervised group (Hybrid Group) performed a resistance training programme two days per week during 8 weeks, combined with the home-based physical activity plan. All supervised training sessions were conducted by a physiotherapist (See Supplementary Material S1: Full description of the supervised resistance training programme and home-based physical activity plan). The other group only participated in the home-based physical activity plan for 8 weeks (Home Group). All participants were contacted by telephone once a week to monitor adherence.

### 2.4. Variable and Outcomes: Type and Measurement

Outcome measures were recorded at baseline and at the end of the study intervention period for both groups.

#### 2.4.1. Primary Variable and Outcome

##### Health-Related Quality of Life

Health-related quality of life (HRQoL) was assessed using the European Organisation for Research and Treatment of Cancer Quality of Life Questionnaire (EORTC QLQ-C30) [15]. This questionnaire has been validated in Spanish and includes 30 items, 24 of which are grouped into five functional scales (physical, role, emotional, cognitive, and social), three symptom scales (fatigue, pain, and nausea/vomiting), and one global health status scale. Each domain within the QLQ-C30 can achieve a maximum score of 100 points. For functional domains, higher scores reflect better HRQoL, whereas for symptom domains, higher scores correspond to poorer quality of life. The minimum clinically important difference (MCID) for the EORTC QLQ-C30 is generally considered to be 10 points for both functional and symptom scales, which represents a clinically meaningful improvement or deterioration in patient-reported outcomes [16].

#### 2.4.2. Secondary Variable and Outcomes

##### Anxiety and Depression

Anxiety and depression were evaluated using the Hospital Anxiety and Depression Scale (HADS), a validated and reliable instrument for various populations [17]. The HADS includes 14 items: seven related to anxiety (subscale A) and other seven to depression (subscale D), each scoring from 0 to 3, resulting in two separate scores. The questionnaire has been validated in Spanish. For the HADS instrument, previous validation studies report a minimum clinically important difference of approximately 1.5–1.7 points for each subscale, reflecting a meaningful change in anxiety or depression severity [18].

##### Sleep Quality

Sleep quality was assessed using the Minimal Insomnia Symptom Scale (MISS), a brief questionnaire developed to identify insomnia symptoms [19]. The scale includes three items, each rated on a five-point Likert scale from 0 (no problems) to 4 (severe problems). Total scores range from 0 to 12, with higher scores reflecting greater sleep disturbances. The MISS has demonstrated strong reliability and validity, particularly in older populations. In the sample, the reliability coefficient was 0.79. The questionnaire has been validated in Spanish. However, no minimum clinically important difference (MCID) or minimum detectable change (MDC) has been defined in the literature. Therefore, changes in MISS scores are reported descriptively without a predefined threshold for clinical relevance.

##### Inflammatory Parameter

Systemic inflammation was assessed by determining serum levels of C-reactive protein (CRP). Venous blood samples were obtained from all participants. Blood was drawn from the antecubital vein using sterile vacutainer systems and collected into serum separator tubes. Samples were allowed to clot at room temperature for approximately 30 min and then centrifuged at 3000 rpm for 10 min to separate the serum. The supernatant was carefully aliquoted and stored at −80 °C until biochemical analysis. Serum CRP concentrations were quantified using a high-sensitivity immunoturbidimetric assay on an automated clinical chemistry analyser, following the manufacturer’s protocol. All measurements were performed in duplicate, and internal quality controls were applied to ensure analytical precision and reliability. CRP concentrations were expressed in milligrams per litre (mg/L) and used as an indicator of the participants’ inflammatory status.

### 2.5. Sample Size and Randomization

Details on sample size calculation are described in the research protocol [20]. The estimated sample size was 36 participants. Considering a 20% dropout rate based on a previous study [11], the required number of participants was 44, i.e., 22 in each group.

During the randomization process, Microsoft Excel 2020 was used to create a sequence of random numbers, each corresponding to a participant. Participants were individually assigned a unique number from this sequence. Those who received odd numbers were allocated to the Hybrid Group, whereas those with even numbers were assigned to the Home Group. This approach promotes a fair and random distribution of participants between groups, helping to reduce bias and strengthen the reliability of trial’s outcomes.

### 2.6. Blinding

Given the nature of the study, it was not possible to blind the participants to the intervention. However, the evaluators and the statistician were blinded to group allocation, with the latter performing the analysis independently and remaining unaware of the group assignments.

### 2.7. Statistical Methods

Means and standard deviations were calculated for quantitative sociodemographic variables and percentages for qualitative variables. Normality of variables was tested using the Shapiro-Wilks test. The *t*-test or Mann-Whitney U test was used depending on the distribution of the data and the presence of outliers. The Chi-squared test was carried out to examine the association between sociodemographic variables and intervention groups. The median and its confidence intervals [21] were calculated for subscales and scales of the QLQ-C30, HADS and MISS instruments in each intervention groups. Median was used as a descriptive statistic because the normality assumption was not met in the scales of the different instruments and also because the variability present within each intervention group was large. The difference between the pre- and post-intervention scores was used as a measure of change in the different subscales or scales. The intragroup analysis was performed using the Wilcoxon test (see Supplementary Material S2). The Mann-Whitney U test was used to analyze both the difference in the pre and pre-post measures between the intervention groups. The difference between the medians (Hybrid Group-Home Group) was used as an estimate of the magnitude of change in each subscale or scale of the instruments. The rank-biserial correlation derived from the Mann-Whitney U-test [22] was calculated as a standardized effect, which allows the magnitude of the effect to be compared between the different subscales or scales. This effect size ranges from −1 to 1 where absolutes values below 0.125 are low, between 0.125 and 0.465 are moderate and above 0.465 are larger [23]. The significance level used was 0.05 and the confidence intervals were calculated with a confidence level of 95%. Statistical analyses were performed using Jamovi v.2.6 software [24].

## 3. Results

**Figure 1:**
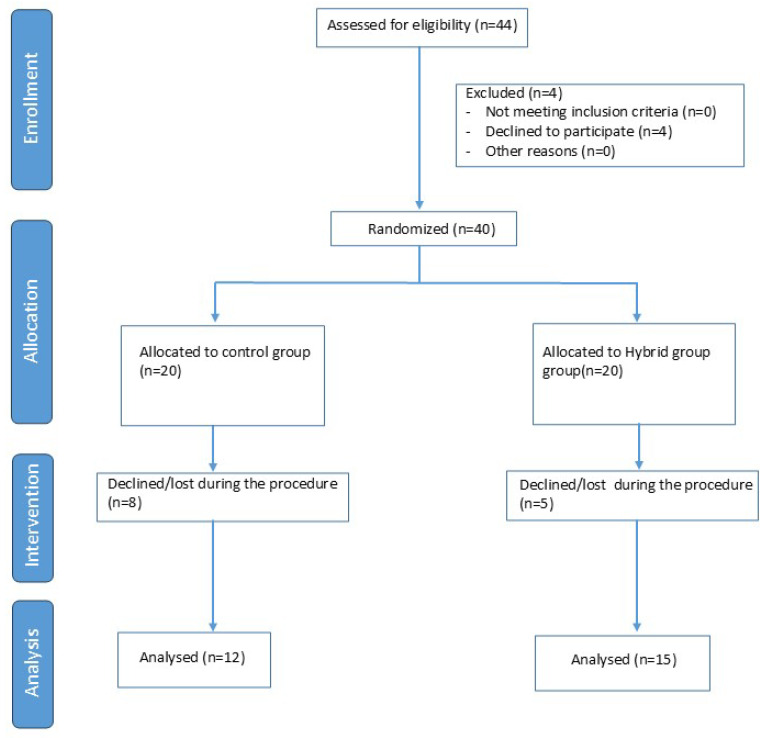
Diagram Flow chart.

Of the 44 participants initially enrolled, 40 were randomised, as 4 ultimately declined to participate. Of the 40 randomised subjects, 27 were included in the data analysis, with 12 subjects in the Home Group and 15 subjects in the Hybrid Group. The evaluation and selection process is described in Figure 1.

### 3.1. Sociodemographic Information

The demographic characteristics of the two groups are detailed in Table 1. The mean age was comparable between the Hybrid Group (59.66  ±  8.70 years) and the Home Group (61.66  ±  13.61 years), with no statistically significant difference (*p*  =  0.647). Similarly, the mean BMI was 25.46  ±  4.39 kg/m^2^ in the Hybrid Group and 24.48  ±  4.28 kg/m^2^ in the Home Group, with no significant difference observed between them (*p*  =  0.568).

Intragroup pre–post analyses were also performed for all variables. As shown in Supplementary Material S2, no statistically significant within-group differences were observed in either the Hybrid Group or the Home Group.

### 3.2. Changes in HRQoL

The intervention groups were homogeneous with regard to pretest measures on all subscales of the QLQ-C30. Statistically significant differences were found between the experimental groups for the pre-post differences on the fatigue and constipation subscales of the QLQ-C30 (Table 2). In the subscale financial impact, all participants answered the same (0) because the treatment they received is part of the public health system in Spain.

**Figure 2:**
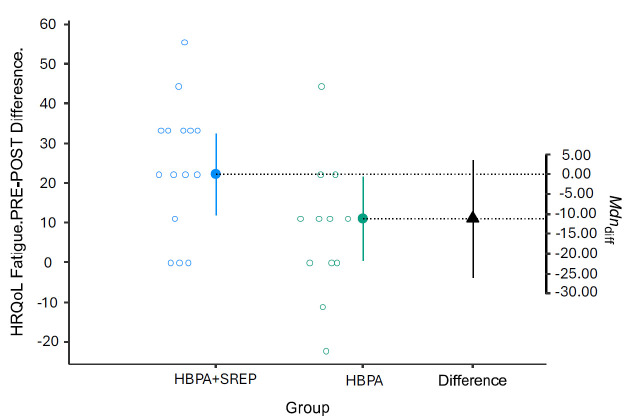
Pre-post changes in the QLQ-C30 fatigue subscale. HRQoL = Health-Related Quality of Life; HBPA + SREP = Hybrid Group; HBPA = Home Group. HBPA = Home Group. The unfilled blue and gray dots represent the pre-post differences for individuals in the intervention and control groups, respectively. The dotted line is the projection on the axis of differences of the median of the differences of the individuals in the intervention group (filled blue dot) and the control group (filled green dot). The filled black triangle is the difference between the previous medians.

**Figure 3:**
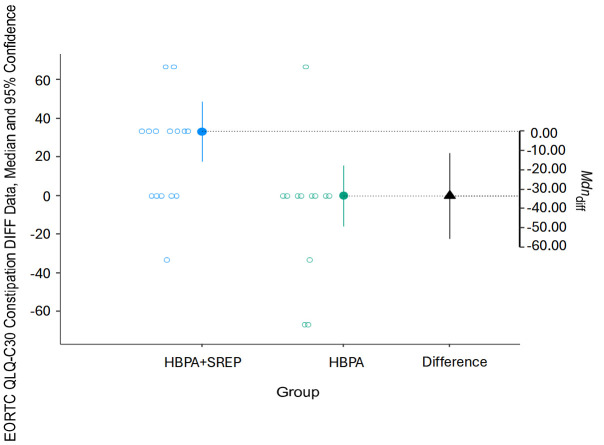
Pre-post changes in the QLQ-C30 constipation subscale. HBPA + SREP = Hybrid Group; HBPA = Home Group. The unfilled blue and gray dots represent the pre-post differences for individuals in the intervention and control groups, respectively. The dotted line is the projection on the axis of differences of the median of the differences of the individuals in the intervention group (filled blue dot) and the control group (filled green dot). The filled black triangle is the difference between the previous medians.

For both subscales, the intervention group showed better results due to lower scores compared to pretest scores (Figure 2 and Figure 3).

### 3.3. Changes in Anxiety, Depression and Sleep Quality

No significant differences were found in the pre-post changes between the groups in relation to the HADS and MISS instruments (Table 3 and Table 4). Effect sizes were low to moderate.

### 3.4. Changes in Inflammation Parameters

No significant differences were found in the pre-post changes between the groups in relation to the inflammation parameter (Table 5). Effect sizes were low to moderate.

### 3.5. Adherence and Adverse Events

Overall adherence to the interventions was excellent, exceeding 80% in all cases, and no adverse events related to physical exercise were reported in any participant

## 4. Discussion

The aim of this study was to compare the effects of a home-based physical activity plus a supervised eight-week resistance exercise programme versus the home-based physical activity during 8 weeks on quality of life, anxiety, depression, and sleep quality in patients with CRC undergoing active treatment with chemo- and/or immunotherapy. Our findings showed that the addition of supervised resistance training led to significant improvements in fatigue and constipation, whereas no differences were observed for other quality-of-life domains, anxiety, depression, or sleep quality. Overall adherence to the interventions was excellent, exceeding 80% in all cases, and no adverse events related to physical exercise were reported in any participant

The improvements in fatigue and constipation observed in our study are clinically relevant, with both exceeding the MCID threshold of 10 points on the EORTC QLQ-C30. This indicates that the observed differences represent not only statistical but also meaningful clinical improvements in symptom burden. The effect sizes for these domains were moderate, suggesting that resistance exercise may provide tangible benefits even within a relatively short intervention period. These results are in line with previous reports showing that exercise, particularly when resistance or combined modalities are employed, can reduce cancer-related fatigue and improve gastrointestinal function. with regard to quality of life, our findings partially align with those of Christensen et al. [25], who implemented a 12-week aerobic programme and reported no significant changes in quality of life. Similar results were reported by Pinto et al. [26] and Hawkes et al. [27] who conducted aerobic interventions lasting 12 weeks and 6 months, respectively. These findings suggest that, even with longer durations than that used in the study, improvements in quality of life are not always observed when the intervention relies exclusively on aerobic training. Conversely, Kim et al. [28] conducted a study employing a 12-week aerobic protocol and reported improvements in both quality of life and mental health—outcomes that contrast with our findings regarding anxiety and depression. Although disappointing at first glance, this finding is consistent with several trials that also reported limited psychological improvements following short-term interventions. Potential explanations include the relatively short duration of the programme, which may not have been sufficient to influence psychosocial variables, and the small sample size, which may have limited statistical power. Moreover, the modality of exercise may play a role: while the protocol focused exclusively on resistance training, some studies using aerobic or combined interventions have reported broader psychological benefits. It is plausible that improvements in emotional wellbeing may require either longer exposure, multimodal training, or complementary psycho-oncological interventions. Another factor to consider is that participants in this trial were undergoing active systemic therapy, which is often associated with fluctuating symptoms, treatment-related distress, and uncertainty. These clinical circumstances may have overshadowed potential improvements in mental health outcomes and sleep. Future studies should evaluate whether exercise programmes of longer duration, higher intensity, or combined with psychosocial support may yield more robust benefits for anxiety, depression, and sleep quality.

Regarding fatigue—one of the domains in which our study observed statistically significant improvements—the results are consistent with those of Bourke et al. [29], who reported substantial reductions in fatigue following a 12-week combined aerobic and resistance training programme. This suggests that resistance training, even in isolation, may have a direct and beneficial effect on fatigue levels in patients with cancer. Similarly, Courneya et al. [30], in a 16-week aerobic exercise intervention, found no significant differences in quality of life, fatigue, anxiety, or depression. These results support the idea that neither the duration of the intervention nor the use of aerobic exercise alone necessarily guarantees clinically or statistically significant improvements in these domains. Such evidence underscores the potential relevance of the training approach applied in the study, characterized by its specific focus on resistance training and its potential effectiveness in addressing symptoms such as fatigue and constipation.

Additionally, upon reviewing recent scientific literature, we identified a study by Dhawan et al. [31], which reported improvements in quality of life following a 10-week intervention incorporating stretching and balance-focused activities. While not directly comparable to our protocol due to differences in both exercise modality and participant characteristics, these findings reinforce the notion that alternative, non-exclusive training approaches may also contribute positively to quality-of-life outcomes. This highlights the importance of developing individualised clinical protocols tailored to the specific needs and capacities of each patient.

Although no significant changes in anxiety, depression, or sleep quality were found in our cohort, similar findings have been reported in previous randomized trials with patients with CRC and other solid tumors. Courneya et al. [12] and Bourke et al. [29] observed improvements in physical outcomes but not in mood or sleep following 12- to 16-week exercise interventions in CRC survivors. Zimmer et al. [11] and Brown et al. [32] also found limited effects on mental health parameters, despite significant improvements in physical aspects and fatigue. In contrast, Kim et al. [28] reported that home exercise improved depressive symptoms and overall quality of life, suggesting that psychosocial outcomes may be more sensitive in the post-treatment or survival phases than during active chemotherapy or immunotherapy, when symptom burden and treatment-related uncertainty are greater. Furthermore, a systematic review conducted by da Silva Bezerra et al. [13] concluded that, although exercise consistently improves quality of life and reduces fatigue in CRC, the results for anxiety, depression, and sleep remain inconclusive, likely due to heterogeneity in intervention duration, intensity, and patient clinical status.

Physical exercise is recognised as one of the main non-pharmacological strategies for managing comorbidities and treatment-related adverse effects in individuals with cancer [32]. However, further research is required to determine the minimum intervention duration necessary to achieve improvements in quality of life, anxiety, depression, or sleep quality. Our findings complement existing evidence showing that aerobic interventions alone often fail to produce consistent improvements in quality of life, while multimodal or resistance-based approaches appear more promising. Collectively, this reinforces the importance of tailoring exercise prescriptions according to modality, duration, and the specific symptom profile of patients.

### 4.1. Implications of the Study

Overall, the results contribute to the growing body of evidence supporting the efficacy of resistance-based exercise programmes in improving specific symptoms, such as fatigue and constipation, in individuals undergoing cancer treatment. Given the relatively short duration of the intervention, it is plausible that a longer exercise protocol could yield broader benefits. This has important implications for clinical practice, particularly in settings where long-term adherence to structured interventions may pose a challenge.

### 4.2. Limitations and Future Directions

The modest sample size and unexpected attrition reduced statistical power and may have led to type II errors. The eight-week intervention period was relatively short, potentially limiting the ability to detect changes in psychological variables. In addition, the inflammatory profile was limited to CRP assessment. Although this provides an initial biological reference, the absence of additional cytokines such as IL-6 and TNF-α restricts the capacity to explore mechanistic pathways. Future studies should include these biomarkers to better elucidate the anti-inflammatory effects of resistance exercise in this population. The lack of follow-up on the changes obtained at 12 and 24 weeks is another limitation. The absence of blinding and the use of a simple randomisation procedure may have introduced bias. In addition, adherence and safety data were not systematically collected, precluding assessment of feasibility and tolerability. Losses during follow-up further restricted generalisability.

Future trials should aim to:

Evaluate longer-duration resistance or multimodal exercise programmes to determine whether broader effects on quality of life and mental health can be achieved.

Incorporate more sensitive measures of psychological wellbeing and sleep quality to capture small changes.

Explore strategies to enhance adherence and monitor safety, particularly in patients undergoing intensive systemic therapy.

Investigate personalised protocols tailored to treatment stage, symptom burden, and patient preferences.

## 5. Conclusions

In this pilot randomized clinical trial, adding a supervised resistance exercise programme to a home-based physical activity plan resulted in improvements in key symptoms commonly experienced by patients with colorectal cancer undergoing chemotherapy and/or immunotherapy, particularly fatigue and constipation. No meaningful changes were observed in anxiety, depression, or sleep quality. Overall, the findings indicate that supervised resistance training is a safe and feasible strategy that may enhance selected aspects of quality of life during active treatment. Larger and longer-term trials, including broader biomarker profiling, are warranted to confirm these preliminary results and to better understand the mechanisms underlying these benefits.

## Figures and Tables

**Table 1:** Sociodemographic characteristics.

Variable	Home Group (*n* = 12)		Hybrid Group (*n* = 15)	
	No.	%	No.	%
Age (years) (*p*-value = 0.647)				
Mean	61.66		59.66	
SD	13.61		8.70	
Gender (*p*-value = 0.381)				
Male	6	50	10	66
Female	6	50	5	34
Weight (kg) (*p*-value = 0.578)				
Mean	67.5		70.4	
SD	11.61		12.81	
BMI (kg/m^2^) (*p*-value = 0.568)				
Mean	24.48		25.46	
SD	4.28		4.39	
Cancer type				
Colon	12	100	15	100
Rectal	0	0	0	0
Cancer Stage (*p*-value = 0.241)				
II	1	8	2	13
III	7	59	6	40
IV	4	33	7	47
Number of Cycles (*p*-value = 0.657)				
Median	4		4.5	
IR	5.50		7.25	
Type of treatment (*p*-value = 0.657)				
CT	9	75	8	54
IO	0	0	1	6
CT + IO	3	25	6	40
Months of evolution (*p*-value = 0.250)				
Median	6		5.50	
IR	28.5		5	

Note. SD = Statistical Deviation; IR = Interquartile Range; CT = Chemotherapy; IO = Immunotherapy; BMI = Body Mass Index.

**Table 2:** Changes in health-related quality of life (HRQoL).

Variable	Median Difference (95%CI)	Effect Size	*p*-Value
Physical	6.67 (−9.02 to 22.36)	0.21	0.358
Role	16.67 (−11.64 to 44.98)	0.21	0.319
Cognitive	0.00 (−17.59 to 17.59)	0.22	0.307
Emotional	0.00 (−11.07 to 11.07)	−0.06	0.824
Social	0.00 (−11.07 to 11.07)	0.18	0.382
Global health status	−8.33 (−22.49 to 5.82)	−0.02	0.941
Fatigue	−11.11 (−22.49 to 5.82)	−0.47	0.040
Nausea	0.00 (−11.07 to 11.07)	−0.02	0.954
Pain	0.00 (−17.59 to 17.59)	−0.07	0.777
Dyspnoea	0.00 (−15.79 to 15.79)	−0.17	0.341
Sleep disturbance	0.00 (−15.51 to 15.51)	−0.17	0.382
Appetite Loss	0.00 (−31.58 to 31.58)	−0.07	0.739
Constipation	−33.33 (−54.46 to −11.20)	−0.53	0.015
Diarrhoea	0.00 (−31.58 to 31.58)	0.08	0.638

**Table 3:** Changes in anxiety and depression.

Variable	Median Difference (95%CI)	Effect Size	*p*-Value
HADS-Anxiety	1.00 (−1.84 to 3.84)	0.09	0.694
HADS-Depression	−0.50 (−3.23 to 2.23)	−0.02	0.941
HADS-Total	1.50 (−2.30 to 5.30)	0.08	0.732

**Table 4:** Changes in sleep quality.

Variable	Median Difference (95%CI)	Effect Size	*p*-Value
Sleep quality	−0.5 (−3.01 to 2.01)	−0.33	0.149

**Table 5:** Changes in inflammation parameters.

Variable	Median Difference (95%CI)	Effect Size	*p*-Value
CRP	−0.175 (−0.4 to 0.0497)	−0.31	0.179

## Data Availability

All data has been given in the manuscript and Supplementary Files. The data-set can be obtained from the corresponding author on request.

## Supplementary Materials

The following supporting information can be downloaded at: https://www.mdpi.com/article/10.3390/curroncol32120651/s1, Suplementary S1: Supervised resistance training programme; Suplementary S2: The results of intragroup analysis using the Wilcoxon test.
