# Supplementary material for: Effects of Resistance Exercise on Quality of Life, Anxiety, Depression, Sleep Quality and Inflammatory Parameters in Patients with Colorectal Cancer Undergoing Active Treatment: A Pilot Randomized Clinical Trial
Source: Curr Oncol. 2025 Nov 21;32(12):651. doi: 10.3390/curroncol32120651 (PMC12731986; doi:10.3390/curroncol32120651)
Supplement: Supplementary file 1 [file curroncol-32-00651-s001.zip › curroncol-3980470-supplementary.pdf]

Supplementary Material S1

SUPERVISED RESISTANCE TRAINING PROGRAMME

SESSION 1

1.- Warm-up (10 minutes)

- 2 minutes: Walk in circles taking advantage of the width of the room incorporating mobility exercises.
- 20 seconds: Just walk.
- 20 seconds: Shoulder circles forward.
- 20 seconds: Shoulder circles backward.
- 20 seconds: Alternating knee lifts to chest.
- 20 seconds: Walk on tiptoes.
- 20 seconds: Hip separation walking.
- 2 minutes: Single-leg balance with slight knee and hip flexion, 1 minute on each leg.
- 1 minute: Wall/floor push-ups.
- 1 minute: Wall sit.
- 2 minutes: Skipping.
- 2 minutes: Ball toss in pairs or against a wall.

2.- Resistance Training

- Normal squat.
- Romanian deadlift.
- Abdominal plank (30 seconds).
- Bench press with barbell.
- Bicep curls with dumbbells.
- Dumbbell rows.

**There will be 6 stations, and participants will rotate from one to the next until completing all 6 exercises. They will do 3 sets of 12 repetitions at 70% of 1RM. Rest 30 seconds between stations and at the end of each complete set, rest for 90 seconds.*

3.- Cool-down

- 3 minutes: Slow-paced walking with diaphragmatic breathing.
- 1 minute: Quadriceps stretch.
- 1 minute: Hamstring stretch.
- 1 minute: Calf stretch.
- 1 minute: Bicep stretch.
- 1 minute: Shoulder stretch.
- 2 minutes: Seated cervical mobility exercises.

SESSION 2

1.- Warm-up (10 minutes)

- 2 minutes: Walk in circles taking advantage of the room's width incorporating mobility exercises.
- 20 seconds: Just walk.
- 20 seconds: Shoulder abduction/adduction in transverse plane.
- 20 seconds: Alternating shoulder flexion/extension.
- 20 seconds: Leg lifts with straight knees alternately.
- 20 seconds: Heel walks.
- 20 seconds: Jump in place.
- 2 minutes: Ankle proprioception on single-leg support, 1 minute on each leg.
- 1 minute: Knee lift with contralateral arm raise.
- 1 minute: Bodyweight squats/sit and stand from a chair.
- 2 minutes: Step-up with contralateral knee raise.
- 2 minutes: Boxing (punching forward with alternate arms at a slow pace).

2.- Resistance Training

- Lunge.
- Glute bridge with barbell/kettlebell.
- Abdominal crunches.
- Dumbbell bench press.
- Shoulder press.
- Sit-to-stand jumps.

**There will be 6 stations, and participants will rotate from one to the next until completing all 6 exercises. They will do 3 sets of 12 repetitions at 70% of 1RM. Rest 30 seconds between stations and at the end of each complete set, rest for 90 seconds.*

3.- Cool-down

- 3 minutes: Slow-paced walking with diaphragmatic breathing.
- 1 minute: Quadriceps stretch.
- 1 minute: Glute stretch.
- 1 minute: Adductor stretch.
- 1 minute: Hamstring stretch.
- 1 minute: Shoulder stretch.
- 2 minutes: Seated cervical mobility exercises.

SESSION 3

1.- Warm-up (10 minutes)

- 2 minutes: Walk in circles taking advantage of the room's width incorporating mobility exercises.
- 20 seconds: Just walk.
- 20 seconds: Elbow flexion/extension.
- 20 seconds: Shoulder abduction/adduction.
- 20 seconds: Hip flexion + abduction.
- 20 seconds: Walking lunges.
- 20 seconds: Side steps.
- 2 minutes: Lateral step-ups, 1 minute on each leg.
- 1 minute: Close-grip push-ups on wall/floor.
- 1 minute: Bodyweight alternating lunges in place.
- 2 minutes: Jumping jacks.
- 2 minutes: Bodyweight glute bridges.

2.- Resistance Training

- Wide stance squat.
- Deadlift.
- Barbell rows.
- Barbell bicep curls.
- Dumbbell lateral raises.
- Single-leg box step-ups.

**There will be 6 stations, and participants will rotate from one to the next until completing all 6 exercises. They will do 3 sets of 12 repetitions at 70% of 1RM. Rest 30 seconds between stations and at the end of each complete set, rest for 90 seconds.*

3.- Cool-down

- 3 minutes: Slow-paced walking with diaphragmatic breathing.
- 1 minute: Quadriceps stretch.
- 1 minute: Glute stretch.
- 1 minute: Hamstring stretch.
- 1 minute: Bicep stretch.
- 1 minute: Shoulder stretch.
- 2 minutes: Seated cervical mobility exercises.

SESSION 4

1.- Warm-up (10 minutes)

- 2 minutes: Walk in circles taking advantage of the room's width incorporating mobility exercises.
- 20 seconds: Just walk.
- 20 seconds: Squat and stand up as you walk.
- 20 seconds: Trunk rotations with shoulder push.
- 20 seconds: Shoulder raises.
- 20 seconds: Side lunges.
- 20 seconds: Walk backward.
- 2 minutes: Sit and stand from a chair.
- 1 minute: Theraband elbow flexion.
- 1 minute: Calf raises and holds statically.
- 2 minutes: Jumping in place.
- 2 minutes: Shoulder circumduction with light weight.

2.- Resistance Training

- Lateral lunge.
- Deadlift.
- Incline bench chest fly with dumbbells.
- Supine tricep press.
- Front shoulder raises with plate.
- Jump squats.

**There will be 6 stations, and participants will rotate from one to the next until completing all 6 exercises. They will do 3 sets of 12 repetitions at 70% of 1RM. Rest 30 seconds between stations and at the end of each complete set, rest for 90 seconds.*

3.- Cool-down

- 3 minutes: Slow-paced walking with diaphragmatic breathing.
- 1 minute: Quadriceps stretch.
- 1 minute: Glute stretch.
- 1 minute: Tricep stretch.
- 1 minute: Chest stretch.
- 1 minute: Shoulder stretch.
- 2 minutes: Seated cervical mobility exercises.

HOME-BASED PHYSICAL ACTIVITY PLAN

The following exercises should be performed three days per week (each day you will do the five exercises assigned to day one, day two, or day three). These sessions should not coincide with the days of physiotherapist-led training.

Each exercise should be performed for 1 minute, followed by 1 minute of rest before starting the next exercise. You should complete 2 full sets of the 5 exercises. In total, the daily programme will take around 20 minutes.

Additionally, you must walk for 1 hour every day.

DAY 1:

- Sit-to-stand from a chair.
- Lateral lunge.
- Supine cycling (lying on your back).
- Elbow flexion.
- Lateral shoulder raise.

DAY 2:

- Lunge.
- Quadriceps wall sit (isometric hold).
- Glute bridge (lying on your back).
- Frontal shoulder raise.
- Wall push-ups.

DAY 3:

- Tiptoe walking.
- Squats.
- Knee-to-chest raise.
- Abdominal crunches.
- Shoulder press.

*Example: On Tuesday, perform the five exercises from DAY 1; on Wednesday, the five exercises from DAY 2; and on Friday, the five exercises from DAY 3. You must walk for 1 hour every day.

Supplementary Material S2

Variable	Groups	N	Median	LL	UL	*p-value*
			**Functional**			
Physical	Hybrid Group	15	-13.33	-22.64	-4.03	*0.003*
	Home Group	12	-6.67	-19.30	5.97	*0.057*
Role	Hybrid Group	15	-16.67	-32.17	-1.16	*0.006*
	Home Group	12	0.00	-23.69	23.69	*0.102*
Cognitive	Hybrid Group	15	0.00	-7.75	7.75	*0.044*
	Home Group	12	0.00	-15.79	15.79	*0.516*
Emotional	Hybrid Group	15	-8.33	-16.09	-0.58	*0.036*
	Home Group	12	-8.33	-16.23	-0.44	*0.046*
Social	Hybrid Group	15	0.00	-7.75	7.755	*0.063*
	Home Group	12	0.00	-7.90	7.90	*0.500*
Global health status	Hybrid Group	15	0.00	-7.75	7.75	*<0.001*
	Home Group	12	-8.33	-20.18	3.51	*0.002*
			**Sympton**			
Fatigue	Hybrid Group	15	22.22	11.88	32.56	*0.002*
	Home Group	12	11.11	0.58	21.64	*0.130*
Nausea	Hybrid Group	15	0.00	-7.75	7.75	*0.084*
	Home Group	12	0.00	-7.90	7.90	*0.102*
Pain	Hybrid Group	15	0.00	-7.75	7.753	*0.884*
	Home Group	12	0.00	-15.79	15.79	*0.666*

Dyspnoea	Hybrid Group	15	0.00	0.00	0.00	*0.157*
	Home Group	12	0.00	-15.79	15.79	*1*
Sleep Disturbance	Hybrid Group	15	0.00	-15.51	15.51	*0.072*
	Home Group	12	0.00	0.00	0.00	*0.414*
Appetite loss	Hybrid Group	15	0.00	0.00	0.00	*0.157*
	Home Group	12	0.00	-31.58	31.58	*0.832*
Constipation	Hybrid Group	15	33.33	17.83	48.84	*0.011*
	Home Group	12	0.00	-15.79	15.79	*0.40*
Diarroeha	Hybrid Group	15	0.00	0.00	0.00	*0.102*
	Home Group	12	0.00	0.00	0.00	*0.564*

Note. LL= Lower Limit; UL= Upper Limit

Variable	*Groups*	*N*	*Median*	*LL*	*UL*	*p-value*
HADS- Anxiety	Hybrid Group	15	0.000	-2.326	2.33	*0.266*
	Home Group	12	1.000	-1.842	3.84	*0.166*
HADS-Depression	Hybrid Group	15	1.000	-1.326	3.33	*0.776*
	Home Group	12	0.500	-0.921	1.92	*0.533*
HADS- Total	Hybrid Group	15	1.000	-0.861	2.86	*<0.001*
	Home Group	12	2.500	-0.816	5.82	*0.003*

Note. LL= Lower Limit; UL= Upper Limit

Variable	*Groups*	*N*	*Median*	*LL*	*UL*	*p-value*
Sleep Quality	Hybrid Group	15	1.000	-1.326	3.33	*0.014*
	Home Group	12	0.500	-0.447	1.45	*0.328*

Variable	*Groups*	*N*	*Median*	*LL*	*UL*	*p-value*
CRP	Hybrid Group	15	0	-0.181	0.181	*0.999*
	Home Group	12	-0.175	-0.308	0.05	*0.100*

Note. LL= Lower Limit; UL= Upper Limit
